# Genetic Causal Association Between Vitamin E and Depression: A Two‐Sample Mendelian Randomization Study

**DOI:** 10.1002/brb3.71510

**Published:** 2026-05-29

**Authors:** Dehua Zhao, Xiaoqing Long, Jisheng Wang

**Affiliations:** ^1^ Department of Clinical Pharmacy The Third Hospital of Mianyang (Sichuan Mental Health Center) Mianyang Sichuan People's Republic of China

## Abstract

**Background and Purpose:**

Evidence regarding the association between vitamin E and depression is inconsistent. To address this uncertainty, we employed a two‐sample Mendelian randomization (MR) approach to evaluate the potential causal effect of vitamin E on depression risk.

**Methods:**

Genetic associations for vitamin E and depression were obtained from large‐scale, publicly available genome‐wide association studies (GWAS). We applied five complementary MR methods: inverse‐variance weighted (IVW), MR‐Egger, weighted median, simple mode, and weighted mode, with IVW serving as the primary analysis. To evaluate the robustness of our findings, we performed sensitivity analyses including Cochran's *Q* test, the MR‐Egger intercept test, MR‐Pleiotropy Residual Sum and Outlier (MR‐PRESSO), and leave‐one‐out analyses.

**Results:**

In total, nine single‐nucleotide polymorphisms (SNPs) were selected as instrumental variables (IVs). Across all MR methods, the odds ratios (ORs) were statistically non‐significant (*P* > 0.05): IVW (OR = 3.67, 95% CI: 0.85–15.85, *p* = 0.082), MR‐Egger (OR = 28.77, 95% CI: 1.05–786.24, *p* = 0.087), weighted median (OR = 4.44, 95% CI: 0.73–6.97, *p* = 0.105), simple mode (OR = 1.29, 95% CI: 0.08–21.79, *p* = 0.866), and weighted mode (OR = 1.29, 95% CI: 0.08–21.84, *p* = 0.866). The robustness of these null findings was supported by subsequent sensitivity analyses.

**Conclusions:**

The present MR analysis indicated that genetically predicted vitamin E was not causally associated with the risk of depression. However, a non‐linear association cannot be excluded and thus merits further investigation.

## Graphical abstract

The Mendelian Randomization (MR) study was conducted using three core assumptions: (1) Instrumental variables (IVs) are strongly associated with the exposure; (2) IVs are not influenced by any confounding variables; (3) IVs affect the outcome solely through the exposure. The present MR analysis indicated that genetically predicted vitamin E was not causally associated with the risk of depression. Sensitivity analyses validated the reliability of these results.

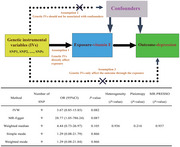

## 1 Introduction

Depression constitutes a major global health concern, affecting over 300 million individuals worldwide across all age groups (Huang et al. 2023). It is associated with reduced productivity, diminished quality of life, and increased risks of mortality and disability (Cavdar et al. 2024). Depression imposes a significant economic burden on healthcare systems. Currently, the early diagnosis of depression remains challenging due to the absence of specific biomarkers (Hu et al. 2024). Furthermore, conventional antidepressant therapy remains ineffective for approximately one‐third of patients with depression (Gonda et al. 2024). Consequently, depression constitutes a major public health challenge that demands more effective interventions.

The precise pathogenesis of depression remains incompletely elucidated. Current evidence supports a multifactorial etiology, encompassing genetic, biological, psychological, and social determinants (Tang et al. 2024). Key biological factors implicated in depression include oxidative stress, the tryptophan catabolite pathway, and inflammatory processes (Wigner et al. 2018). Recent epidemiology studies have shown that dietary elements may benefit individuals with depression (Das et al. 2021). Vitamin E is a fat‐soluble antioxidant that typically protects against various disorders resulting from oxidative damage. Since oxidative stress generates neuron‐harming free radicals linked to depressive symptoms, vitamin E may, in theory, counteract depression by reducing such damage (Vaváková et al. 2015). Previous studies have investigated the association between vitamin E and the risk of depression (Huang et al. 2023; Farhadnejad et al. 2020). A retrospective analysis of 2017–2020 National Health and Nutrition Examination Survey (NHANES) data indicated that higher vitamin E intake (up to 15 mg/day) was associated with a reduced likelihood of depressive symptoms (OR = 0.87, 95% CI: 0.77–0.97, *P* < 0.01), after adjusting for age, race, sex, and income (Huang et al. 2023). In contrast, a cross‐sectional study of 263 female adolescents in Tehran demonstrated no significant association between vitamin E intake and depression within this specific demographic (Farhadnejad et al. 2020). These discrepancies could stem from methodological limitations, including residual confounding, unmeasured factors, and the potential for reverse causality inherent in observational designs. Consequently, the causal relationship between vitamin E and depression remains unclear and merits further investigation.

Mendelian randomization (MR) is an epidemiological approach that leverages genetic variants as instrumental variables (IVs) to infer causality. This design minimizes residual confounding and addresses reverse causation, thereby strengthening causal inference (Hemani et al. 2018). The MR approach is grounded in Mendel's laws of inheritance. The random segregation and independent assortment of alleles during gamete formation mimics the random assignment in controlled trials, thereby largely avoiding the environmental confounding factors that complicate observational epidemiology (Skrivankova et al. 2021, Skrivankova et al. 2021). Given its ability to mitigate confounding bias, strengthen causal inference, and overcome limitations of observational studies, MR has been widely employed to investigate associations between nutritional factors and depression (Hui et al. 2024; Carnegie et al. 2024; Møllehave et al. 2017). The availability of large‐scale genome‐wide association study (GWAS) summary statistics for vitamin E and depression enables well‐powered MR analyses. Leveraging these data, we performed a two‐sample MR study to investigate the potential causal effect of vitamin E on depression risk.

## 2 Methods

### 2.1 Study Design

**FIGURE 1:**
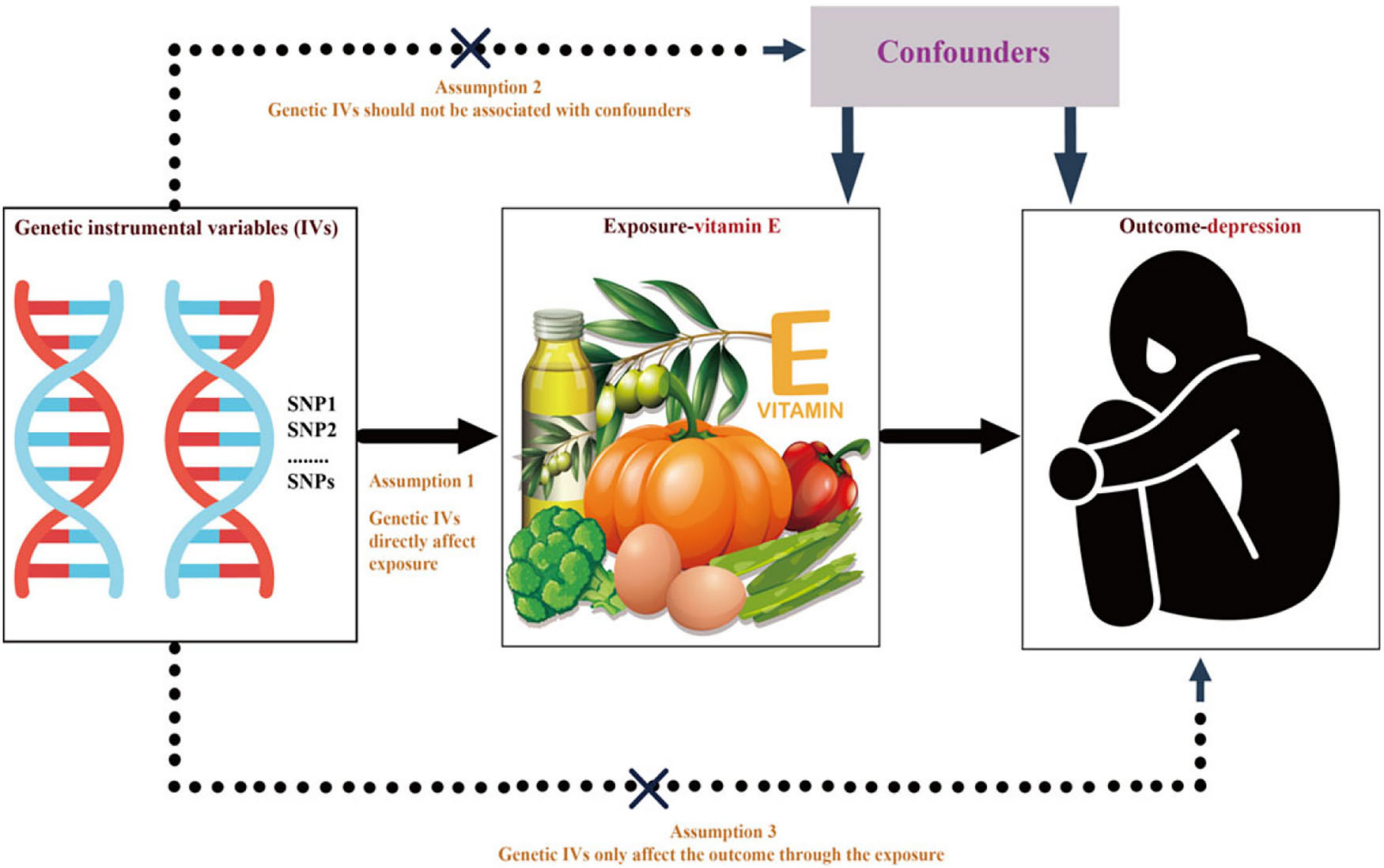
Overview of the study design.

This study adhered to the Strengthening the Reporting of Observational Studies in Epidemiology–Mendelian Randomization (STROBE‐MR) guidelines. We leveraged publicly available GWAS summary data to perform a two‐sample MR analysis investigating the causal effect of vitamin E on depression risk. The analysis was designed in accordance with the three core assumptions of MR (Rosoff et al., 2021): (1) The IVs are strongly associated with the exposure; (2) IVs are independent of confounders; and (3) IVs influence the outcome exclusively through the exposure, not via alternative pathways. The overall study design was outlined in Figure 1.

### 2.2 Data Sources

Genetic instruments for vitamin E were derived from a GWAS meta‐analysis of 462,933 individuals of European ancestry. Genetic associations for depression were obtained from a separate GWAS meta‐analysis comprising 170,756 cases and 329,443 controls, also of European ancestry. The characteristics of these GWAS summary datasets were detailed in Table 1.

**TABLE 1:** Details of the GWAS datasets.

GWAS ID	Year	Trait	Consortium	Sample size
ukb‐b‐16288	2018	Vitamin E	MRC‐IEU	462,933
IEU‐B‐102	2019	Major depression	PGC	500,199

Abbreviations: MRC‐IEU, medical research council and integrative epidemiology unit; PGC, Psychiatric Genomics Consortium.

### 2.3 Genetic IVs Selection

To identify valid genetic instruments, we applied stringent selection criteria. Primary selection employed a genome‐wide significance threshold (*P* < 5 × 10^−^^8^) for single‐nucleotide polymorphisms (SNPs) associated with vitamin E. If fewer than three SNPs were available, a secondary, less stringent threshold (*P* < 5 × 10^−^^6^) was applied to ensure an adequate number of instruments for analysis (Wu et al. 2024). We applied a strict threshold of *r* ^2^ = 0.001 within a 10,000 kb window to clump the identified SNPs for linkage disequilibrium (LD) (Rosoff et al. 2021). The strength of the genetic instruments was evaluated using the *F*‐statistic. To mitigate weak instrument bias, SNPs with an *F*‐statistic below the conventional threshold of 10 were excluded from the analysis (Wu et al. 2024). Genetic instruments were removed if they either were unavailable in the outcome dataset or demonstrated an association with the outcome at a genome‐wide significance level (*P* < 1.0 × 10^−^^5^) (Kennedy et al. 2020). We harmonized the exposure and outcome data and excluded any palindromic SNPs with intermediate‐effect allele frequencies (0.40 < effect allele frequency < 0.60). Finally, all candidate SNPs were systematically screened using PhenoScanner V2 to exclude those associated with known potential confounders (Wu et al. 2024).

### 2.4 MR Analysis

A two‐sample MR analysis was conducted with the selected genetic instruments to estimate the causal effect of vitamin E on depression risk, employing the inverse‐variance weighted (IVW) method under a multiplicative random‐effects model as the primary analytical approach (Rosoff et al. 2021; Wu et al. 2024; Kennedy et al. 2020). The IVW method leverages meta‐analysis techniques to combine Wald ratios from individual SNPs. This approach rests on the core MR assumption that IVs influence the outcome solely via the exposure of interest (Rosoff et al. 2021). To complement the primary IVW analysis and assess the robustness of our findings, we employed the following additional MR methods: MR‐Egger, weighted median, simple mode, and weighted mode (Rosoff et al. 2021; Wu et al. 2024; Kennedy et al. 2020). These complementary methods often provide more robust estimates under violations of standard assumptions, albeit at the expense of statistical efficiency. All MR estimates are reported as odds ratios (ORs) with corresponding 95% confidence intervals (CIs).

### 2.5 Sensitivity Analyses

To evaluate the robustness of the primary MR findings, we performed multiple sensitivity analyses. Heterogeneity across IVs was assessed using Cochran's *Q* test, where *P* < 0.05 indicates significant heterogeneity (Wu et al. 2024). Potential horizontal pleiotropy was examined via the MR‐Egger intercept test (*P* < 0.05 deemed significant) (Kennedy et al. 2020) and the MR‐Pleiotropy Residual Sum and Outlier (MR‐PRESSO) method for outlier detection and global pleiotropy testing (global *P* < 0.05 suggests pleiotropy) (Wu et al. 2024; Kennedy et al. 2020). Additionally, a leave‐one‐out analysis was conducted to ensure no single SNP disproportionately drove the overall causal estimate (Rosoff et al. 2021; Wu et al. 2024; Kennedy et al. 2020).

### 2.6 Analytical Tools

All statistical analyses were performed using *R* (version 4.2.2), primarily utilizing the TwoSampleMR package. A two‐sided *p*‐value of less than 0.05 was defined as statistically significant.

## 3 Results

### 3.1 Genetic IVs

**FIGURE 2:**
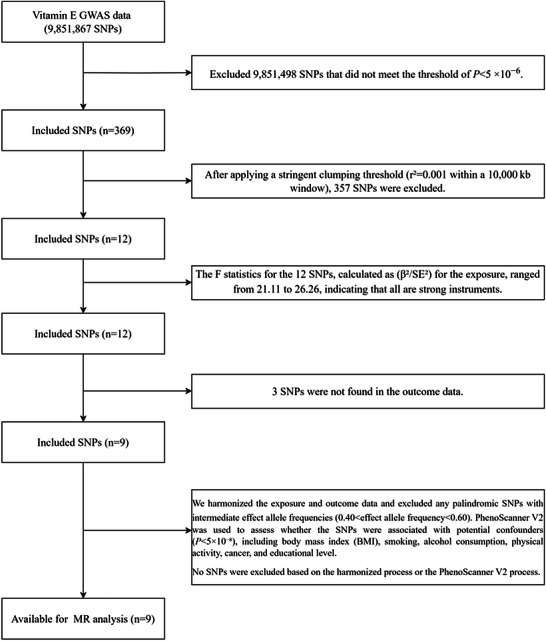
Flow chart of IVs selection.

Given that no SNPs reached genome‐wide significance (*P* < 5 × 10^−8^), a relaxed threshold of *P* < 5 × 10^−6^ was applied for vitamin E, yielding 369 candidate SNPs. Following clumping, 357 SNPs were removed. The remaining variants all had strong *F*‐statistics (range: 21.11–26.26). Three SNPs were absent from the outcome dataset and were excluded. No palindromic SNPs were identified during harmonization, and no further SNPs were removed via PhenoScanner V2. Consequently, 9 SNPs were retained as final instrumental variables for the MR analysis. The complete selection workflow was depicted in Figure 2.

### 3.2 MR Analyses

**FIGURE 3:**
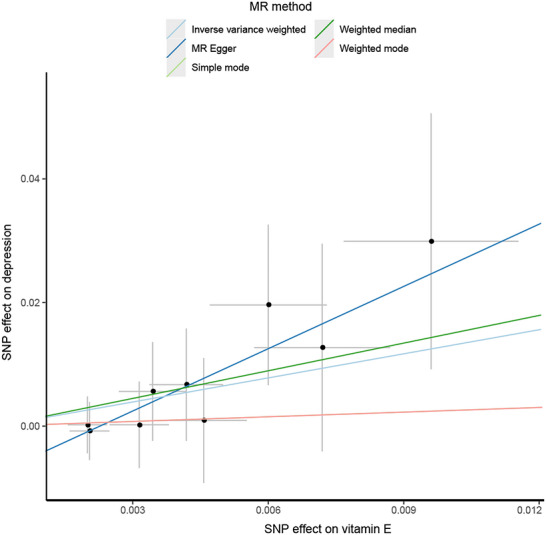
Scatter plot of the MR analysis for the effect of vitamin E on depression. Each point represents a genetic instrument. The slope of the line corresponds to the causal effect estimate derived from different MR methods.

This MR study did not support a causal relationship between vitamin E and depression risk. The primary IVW analysis yielded a non‐significant OR of 3.67 (95% CI: 0.85–15.85, *p* = 0.082). Results from all supplementary methods (MR‐Egger, weighted median, simple mode, and weighted mode) were consistent, showing no statistically significant associations (all *p* > 0.05). Detailed results are presented in Table 2 and visually summarised in the scatter plot (Figure 3).

**TABLE 2:** MR estimates of the causal effects of vitamin E on depression.

Method	Number of SNP	OR (95%CI)	*p*‐value	Heterogeneity (*p*‐value)	Pleiotropy (*p*‐value)	MR‐PRESSO (*p*‐value)
IVW	9	3.67 (0.85–15.85)	0.082	0.936	0.216	0.937
MR‐Egger	9	28.77 (1.05–786.24)	0.087			
Weighted median	9	4.44 (0.73–26.97)	0.105			
Simple mode	9	1.29 (0.08–21.79)	0.866			
Weighted mode	9	1.29 (0.08–21.84)	0.866			

Abbreviations: CI, confidence interval; IVW, inverse‐variance weighted; OR, odds ratio; SNP, single‐nucleotide polymorphisms.

### 3.3 Sensitivity Analyses

**FIGURE 4:**
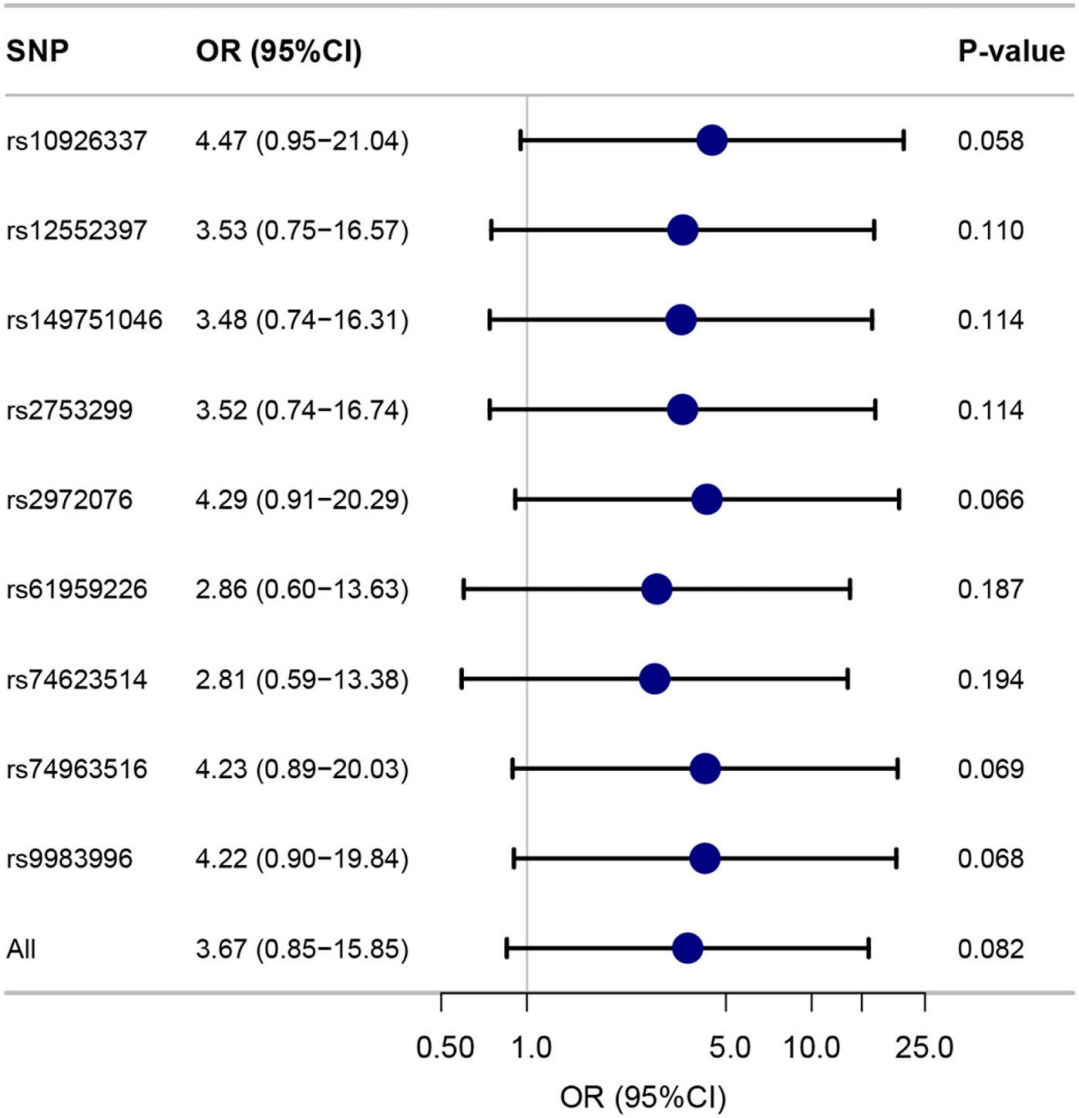
Leave‐one‐out analysis for the MR of vitamin E on depression. The plot displays the IVW estimate (OR with 95% CI) calculated after the sequential exclusion of each SNP. The aggregated estimate using all variants is denoted by “All.”

Cochran's *Q* test indicated no significant heterogeneity (*p* = 0.936). Both the MR‐Egger intercept test (*p* = 0.216) and the MR‐PRESSO test (*p* = 0.937) suggested no evidence of horizontal pleiotropy. Furthermore, leave‐one‐out analysis confirmed that the overall estimate remained stable when any single SNP was omitted (Figure 4).

## 4 Discussion

This study systematically assessed the causal relationship between vitamin E and depression risk using MR analysis based on available summary GWAS data. Our MR analysis found no evidence that vitamin E influences depression risk. Sensitivity analyses confirmed the robustness of this null finding. This study extends the current literature on vitamin E and depression.

Vitamin E, a group of fat‐soluble antioxidants comprising tocopherols and tocotrienols, protects against oxidative damage induced by stress (Das et al. 2021). Oxidative stress is implicated in the pathogenesis of numerous diseases, including depression (Das et al. 2021). It can initiate free radical chain reactions that lead to lipid peroxidation. Vitamin E acts as a crucial chain‐breaking antioxidant, interrupting this process to prevent membrane damage (Manosso et al. 2022). Previous studies have explored the relationship between vitamin E and depression, but the existing evidence remains inconsistent (Jeong et al. 2023; Rubio‐López et al. 2016; Tiemeier et al. 2002; Al‐Fartusie et al. 2019; Kim et al. 2015; Mazloom et al. 2013). An analysis of 2016–2018 Korean NHANES data revealed a stratified association between serum vitamin E and depressive symptoms (Jeong et al. 2023). Specifically, lower serum vitamin E levels were linked to more severe symptoms in younger women and older men, independent of sociodemographic and clinical factors (Jeong et al. 2023). Conversely, no such association was found in younger men or older women. Moreover, a high vitamin E status showed no association with depressive symptoms across all demographic groups (Jeong et al. 2023). A cross‐sectional study of 710 Spanish children (aged 6–9) reported an inverse correlation between dietary vitamin E intake and depressive symptoms (Rubio‐López et al. 2016). In contrast, a study of 263 female adolescents in Tehran found no significant association (Farhadnejad et al., 2020). Furthermore, a population‐based study concluded that low vitamin E levels were not linked to depressive symptoms in the elderly after adjusting for key biological and behavioral confounders (Tiemeier et al. 2002). Evidence from case‐control studies suggested an inverse relationship between vitamin E status and depression. In one study, men with major depressive disorder had significantly lower serum vitamin E levels than healthy controls (*p* < 0.001) (Al‐Fartusie et al. 2019). Similarly, a study of 849 adolescent girls found that higher vitamin E intake was associated with a lower likelihood of depression (Kim et al. 2015). However, a randomized single‐blind trial in patients with type 2 diabetes reported that vitamin E supplementation did not reduce depression risk, indicating that the observed association may not be causal (Mazloom et al. 2013).

The causal interpretation of most existing observational studies on vitamin E and depression is limited by residual confounding and potential reverse causality (Jeong et al. 2023; Rubio‐López et al. 2016; Tiemeier et al. 2002; Al‐Fartusie et al. 2019; Kim et al. 2015). Moreover, evidence from a prior randomized trial is constrained by its small sample size and narrow focus on a specific population (Mazloom et al. 2013). To overcome these issues, the present study employs a two‐sample Mendelian randomization design to assess the causal relationship between vitamin E and depression risk.

Although observational studies have reported an inverse association, our MR analysis found no evidence that genetically predicted vitamin E levels influence depression risk. Depression can alter appetite and dietary intake, making it difficult to discern whether low vitamin E is a cause or a consequence of the disorder (Wu et al. 2023). Furthermore, the complex interactions and antagonisms among nutrients within whole diets complicate the relationship between any single dietary component and depression (Gibson‐Smith et al. 2020). Collectively, these factors may explain the inconsistent findings in prior literature. Our results suggest that vitamin E is not a causal risk factor for depression and that earlier observational associations may be attributable to residual confounding or reverse causation.

Several limitations warrant consideration in interpreting our findings. The selection of genetic instruments using a relaxed significance threshold may raise concerns about weak instrument bias, although all selected variants satisfied standard strength criteria (*F*‐statistic>10). The exclusive focus on European‐ancestry populations limits cross‐ethnic generalizability and calls for validation in other groups. Furthermore, while sensitivity analyses did not detect horizontal pleiotropy, the biological functions of the instrumental SNPs are not fully characterized, leaving some potential for undetected pleiotropic pathways. Finally, the reliance on summary‐level data prevented stratified analyses based on individual‐level characteristics. Despite these limitations, our study has key strengths. The MR approach substantially mitigates confounding and reverse causality, offering a robust method for causal inference. The null findings were consistent across multiple complementary MR methods and were supported by comprehensive sensitivity analyses. Moreover, confining the analysis to a single ancestry group effectively controlled for bias due to population stratification.

## 5 Conclusion

The present MR study did not provide genetic evidence to support a causal effect of vitamin E on depression risk. Nonetheless, a non‐linear association cannot be ruled out. Further validation through larger, well‐designed prospective cohort studies or randomized controlled trials is required to explore this possibility.

## Author Contributions

**Dehua Zhao**: conceptualization, methodology, data curation, software, writing – original draft, writing – review and editing, project administration. **Xiaoqing Long**: methodology, software, data curation. **Jisheng Wang**: data curation, software.

## Funding

The authors have nothing to report.

## Conflicts of Interest

The authors declare no conflicts of interest.

## Data Availability

The data that support the findings of this study are available from the corresponding author upon reasonable request.
